# Health-related quality of life in children after laparoscopic gastrostomy placement

**DOI:** 10.1007/s11136-019-02272-z

**Published:** 2019-08-16

**Authors:** Josephine Franken, Rebecca K. Stellato, Stefaan H.A.J. Tytgat, David C. van der Zee, Femke A. Mauritz, Maud Y.A. Lindeboom

**Affiliations:** 1 Department of Pediatric Surgery, Wilhelmina Children’s Hospital, University Medical Center Utrecht, Lundlaan 6, 3584 EA Utrecht, The Netherlands; 2 Department of Biostatistics, Julius Center for Health Sciences and Primary Care, University Medical Center Utrecht, Heidelberglaan 100, 3584 CX Utrecht, The Netherlands

**Keywords:** Gastrostomy, Quality of life, Laparoscopic gastrostomy, Percutaneous endoscopic gastrostomy

## Abstract

**Introduction:**

A gastrostomy placement (GP) is an established treatment to provide enteral feeding in pediatric patients with feeding difficulties aiming to improve nutritional status and health-related quality of life (HRQoL). The aim of this study was to evaluate HRQoL in children with severe feeding difficulties who have undergone GP.

**Materials and methods:**

A cross-sectional study was performed including 128 patients who had undergone laparoscopic GP (2004–2011). HRQoL was evaluated using the validated Pediatric Quality of Life 4.0 Inventory. Multiple regression analysis was performed to identify predictors of HRQoL.

**Results:**

After a mean follow-up of 4.0 years (interquartile range 2.9–6.2) after GP, mean HRQoL was 53.0 out of 100 (standard deviation 21.1). HRQoL was significantly lower in children with neurologic impairment, with a mean difference of −21.4 points between neurologically impaired and neurologically normal children (*p *< 0.001). HRQoL was also lower in children with cardiac disease (−19.0 points; *p *= 0.01) and in children with a history of previous gastrointestinal surgery (−15.2 points; *p *= 0.03). Feeding through a gastrojejunostomy tube (−33.0 points; *p *= 0.01) and higher age at the time of operation (−1.2 points per year; *p *= 0.03) were also associated with lower HRQoL. GP-related complications requiring reintervention were associated with lower HRQoL, although this association was not statistically significant (*p *= 0.06).

**Conclusions:**

Children with severe feeding difficulty, who have undergone GP, have significantly lower HRQoL compared to a healthy pediatric population. Neurologic impairment, cardiac disease, a history of gastrointestinal surgery, older age, and the need for jejunal feeding through the gastrostomy were predictive of even lower HRQoL.

## Introduction

A gastrostomy placement (GP) is an effective treatment to provide enteral tube feeding in children in need of nutritional support. [1, 2] Previous studies on GP in children have focused primarily on the physical outcomes of patients after operation [3]. Health-related quality of life (HRQoL) is increasingly recognized as an essential component of patient care outcomes. It aims to assess the impact of an illness and its treatment on the dimensions of physical, psychological, and social health [4–8]. However, little is known about HRQoL as a patient care outcome in children undergoing GP, and about the factors influencing HRQoL.

Although laparoscopic gastrostomy is generally regarded as a safe procedure, minor complications such as hypergranulation, gastrostomy site infection, or leakage around the catheter frequently occur. [2] These minor complications can be reason for reintervention or hospital readmission in 16% of patients. [9] Also, some children do not tolerate feeding directly into the stomach and need to be fed through a jejunal tube with a 24-hour continuous feeding scheme. Complications related to GP may therefore have an influence on the HRQoL of patients with a GP.

The main indication for GP in children is feeding difficulty, in most cases caused by neurologic impairment (NI), cardiac disease, or cystic fibrosis. [10] According to two previous studies that investigated HRQoL using the PedsQL in various disease categories, children with NI self-reported the lowest HRQoL out of all possible categories. HRQoL of pediatric patients with cystic fibrosis was less profoundly affected. [11] Children with congenital cardiac disease had HRQoL closest to the healthy pediatric population. [12] Presumably, in our study HRQoL may be profoundly affected by the child’s primary health condition, and can be expected to differ among the various morbidities.

Many children requiring GP have coexistent gastroesophageal reflux (GER), especially those with NI. [13] After GP, symptoms of GER are seen in 25–66% of patients. [14, 15] The prevalence of GER may also have a significant impact on HRQoL. [16].

Few studies performed in children undergoing GP have focused on HRQoL. One study investigated quality of life in children before and after GP, reporting no significant changes. [17]. However, this study did not use validated questionnaires for quality of life assessment. A few other studies focused on the experience of parents of children undergoing GP. [18–20] These studies reported a positive impact of GP on the HRQoL of parents, seen in a decrease in burden of care [19] and an increase in self-reported social functioning, energy, and general health perception. [18] These studies did not report on the HRQoL of the children themselves. To our knowledge, there is a lack of well-designed studies on the HRQoL in children undergoing GP. Where the aforementioned studies did not use validated HRQoL questionnaires, in this study the Pediatric Quality of Life (PedsQL^TM^) 4.0 Generic Core Scales were used. This is a reliable and valid tool for proxy-report of HRQoL by caregivers and a parallel self-report for children. It has been used to assess HRQoL in healthy populations, as well as in children with numerous acute and chronic health conditions. [5–7].

Although most children have little alternative for GP, it is important to understand the population undergoing GP and the consequences of GP itself on the lives of these children. This knowledge can help physicians provide better counseling to caregivers before and after GP. The aim of our study was to evaluate HRQoL in children with severe feeding difficulty who have been treated with GP and to identify predictors (both patient characteristics and gastrostomy-related factors) of HRQoL.

## Patients and methods

A cross-sectional study was performed including all children who had undergone GP at the age of 0–18 years between January 2004 and December 2011 at the Wilhelmina Children’s Hospital, University Medical Center Utrecht (UMCU).

### Surgical procedure

GP was performed laparoscopically under general anesthesia in all pediatric patients. All procedures were performed or supervised by an experienced pediatric surgeon. Operations were performed by 6 different pediatric surgeons.

### Ethical approval and informed consent

This study was submitted to the UMCU Ethics Committee (EC). The EC ruled that the current study did not fall under the Medical Research Involving Human Subjects Act.

### Clinical assessment

Patient characteristics and medical history were derived from the electronic patient records. NI was clinically manifested as psychomotor retardation, epilepsy, microcephaly, spasticity, visual impairment, and/or hypotonia.

At assessment, caregivers of children answered multiple questionnaires. Questionnaires were completed by parents (in ‘proxy-report’) and completed in private. For evaluation of HRQoL, the PedsQL^TM^ 4.0 Generic Core Scales was filled out. The PedsQL^TM^ is subdivided into four age-adjusted questionnaires (ages: 2–4; 5–7; 8–12; and 13–18 years) and a parallel self-report for children (ages: 5–7; 8–12; and 13–18 years). The inventory comprises 23 items. The HRQoL total score is divided into two main health scores: physical health summary score (8 items) and psychosocial health summary score (15 items). The psychosocial health score is reflected by the mean of three domains: emotional functioning (5 items), social functioning (5 items), and school functioning (5 items). Items were reverse-scored and scale scores per domain were computed as the sum of the items divided by the number of items answered. Scale scores were then transformed into a scale from 0 to 100, where higher scores indicate better HRQoL. The PedsQL^TM^ version for the age category 2–4 years is shown in Table 1 as an illustration of HRQoL assessment.

**Table 1:** PedsQL^TM^ on health-related quality of life; age category 13–18 years

Could you tell us to what extent your teenager had trouble with each of these things in the last month? There are no right or wrong answers. Please ask for help if you have any questions	
0 if it was **never** a problem 1 if it was **almost never** a problem 2 if it was **sometimes** a problem 3 If it was **often** a problem 4 if it was **almost always** a problem	
Physical functioning (having trouble with…)	
Walking more than 100 meters Running Doing sports or other physical exercise Heavy lifting Taking a bath or shower independently Having pain Feeling tired	
Emotional functioning (having trouble with…)	
Feeling afraid or scared Feeling sad Feeling angry Having trouble sleeping Being worried about what might happen to him/her	
Social functioning (having trouble with…)	
Getting along with other teenagers Other kids not wanting to be friends with her/him Begin bullied by other teenagers Not being able to do things other teenagers of his/her age can do Being able to keep up with other teenagers	
Functioning at school (having trouble with…)	
Paying attention in class Forgetting things Keeping up with work in class and doing his/her homework Not being able to go to school because he/she is not feeling well Not being able to go to school because he/she had to go to the doctor or hospital	

Data on gastroesophageal reflux were obtained from the gastroesophageal reflux symptom Questionnaire (GSQ). [21] In infants, symptoms assessed were back arching, choking or gagging, hiccups, irritability, refusal to feed, and vomiting or regurgitation. In young children, symptoms assessed were abdominal pain, burping or belching, choking when eating, difficulty swallowing, refusal to eat, vomiting, and regurgitation. In this questionnaire, parents scored the symptoms of their child on a frequency scale (0–7 days a week) and a severity scale (0–7). Patients with at least daily and moderately severe symptoms, or at least weakly and severe symptoms were considered positive for symptomatic GER.

Additional information considering the use of the gastrostomy and complications related to GP was collected with the Gastrostomy Placement-specific questionnaire (Table 2).

**Table 2:** Gastrostomy placement—specific questionnaire

Does your child still have a gastrostomy at the moment? Yes/No	
If not, when was it removed? If yes, does he/she have a Mickey button or a permanent catheter? If yes, is he/she fed on the stomach or on the small intestine?	
Did your child undergo other stomach/small intestine/large intestine operations?	
Date and indication for operation: …	
Did your child undergo endoscopical investigations of the stomach or small intestine?	
Date and indication for investigation: …	
How many times did your child undergo a change of the button or catheter?	
1 × 2 × 3 × 4 × 5 × 5–10 > 10	
Did your child experience any of the following complications?	
Leakage at the gastrostomy site: daily/weekly/monthly/yearly/< yearly	
Spontaneous dislocation of the gastrostomy: 1 × 2 × 3 × 4 × 5 × 5–10 > 10	
Infection at the gastrostomy site: 1 × 2 × 3 × 4 × 5 × 5–10 > 10	
Hypergranulation at the gastrostomy site: 1 × 2 × 3 × 4 × 5 × 5–10 > 10	
Other complications: …	
How do you rate your satisfaction with the gastrostomy on a scale from 0–10?	
Can you elaborate on the feeding schedule of your child?	
Portions scattered during the day (with pump)	
Portions scattered during the day (without pump)	
Continuous drip feeding during the night	
Continuous drip feeding during 24 h	
Did your child use any stomach enhancing medication in the last 3–4 months, for instance, domperidon (Motilium) or erythromycin?	
If yes, what kind of medication? In which dosage	
Did your child use any antacid-inhibiting medication in the last 3–4 months, for instance, omeprazole, esomeprazole, or ranitidine?	
If yes, what kind of medication? In which dosage	

### Statistical analysis

The mean HRQoL was investigated in all subdomains of HRQoL assessment. For the PedsQL^TM^ Generic Core Scales, no normal values are available. The only available data as a reference for normal values are published in a large study performed by Varni et al. including 9500 healthy children, having a mean HRQoL score of 82.70 (± 15.40). [12] The difference in total HRQoL between our sample size and the healthy population from Varni et al. was calculated using a two-sample *t* test with Welch’s correction, which is appropriate for when two samples have unequal variances and unequal sample sizes. Differences in HRQoL scores between independent samples of children (for example, between NI and neurologically normal (NN) children) were calculated using the independent samples *t* test.

To correct for incomplete data on HRQoL scores (in case caregivers did not completely fill out the questionnaire in one or multiple subdomains), we used multiple imputation to create 20 complete datasets. [22] Variable groups were used to predict missing values in the imputation model

Multiple linear regression analysis was performed on the imputed data in order to identify predictors of postoperative total HRQoL. Combined results are presented. Since the sample consisted of 128 patients with complete assessment, the maximum number of independent variables entered into the regression analysis was set at 12. The variables chosen to include in the regression analysis were chosen based on univariate analysis (apart from the variables age and gender as general variables). Variables included were age, gender, follow-up time, NI, cardiac disease, history of previous gastrointestinal surgery, acid exposure time (the percentage of time with pH below 4; AET) on preoperative 24-h pH monitoring, gastroesophageal reflux (GER) symptoms, jejunal (versus gastric) feeding, and postoperative return to the operating room. The influence of the independent variables in the prediction of postoperative HRQoL is represented by the mean difference with 95% confidence intervals (95% CI).

Statistical analysis was performed using SPSS 22.0 statistical package (IBM, USA). Statistical significance was defined by *p* values of less than 0.05.

## Results

Three hundred patients had undergone GP between January 2004 and December 2011. Out of these patients, 150 patients and/or their caregivers (50.0%) agreed to participate in the current study. Median age of these children at the time of surgery was 2.7 years (interquartile range (IQR) 1.4–6.0; complete range 22 days–16.7 years). Median age at the time of follow-up was 7.3 (IQR 4.8–11.9). Median follow-up time between GP and HRQoL assessment was 4.0 years (2.9–6.2).

**Fig. 1:**
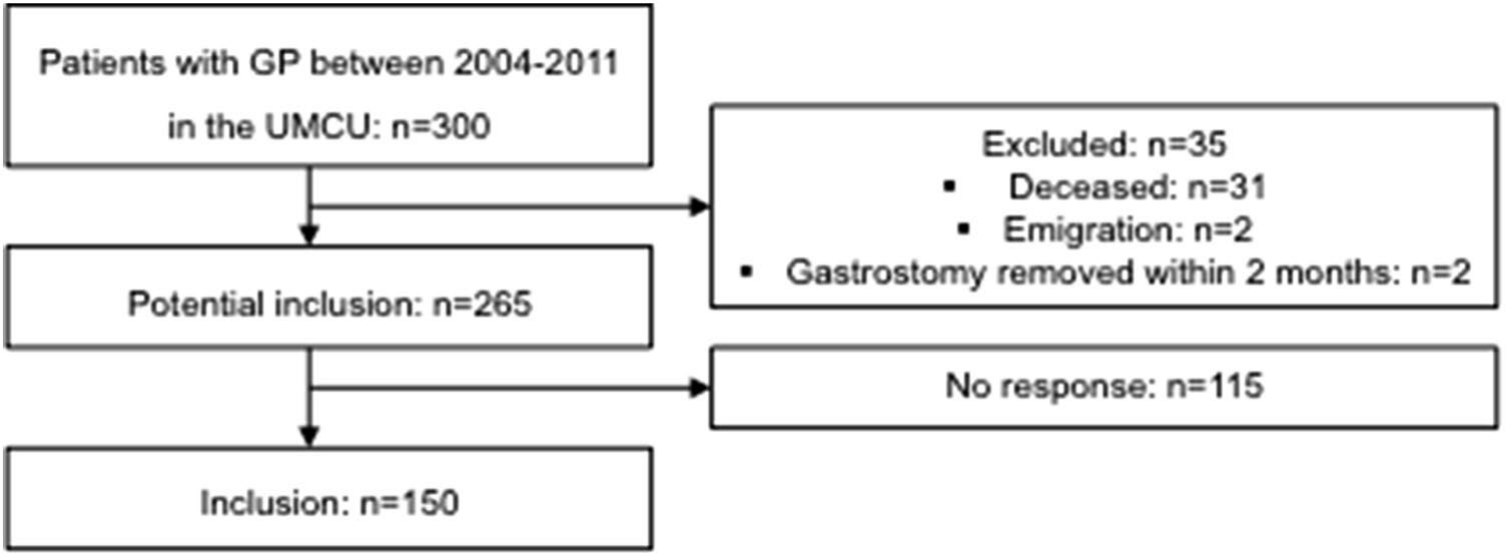
Flowchart of patient inclusion. *GP* gastrostomy placement, *UMCU* University Medical Center Utrecht

Figure 1 depicts an overview of patient inclusion. Out of 150 patients included, 22 caregivers of children (14.7%) did not completely fill out the PedsQL^TM^ questionnaire in one or multiple subdomains of HRQoL. In 13 out of 22 patients, caregivers were unable to answer the questions related to ‘functioning at school/day care.’ The other nine patients also had, in addition to this domain, missing values in the domains of emotional functioning and/or social functioning. The reason for missing values was in all cases severity of the underlying disease.

### Patient characteristics

Patient characteristics are described in Table 3 for both responders and non-responders. The main underlying pathologies were NI (70.7%), cystic fibrosis (11.3%), and cardiac disease (4.7%). Characteristics of non-responders are also given. During follow-up, 26 patients died because of causes unrelated to gastrostomy. Causes of death were deterioration of neurologic disease (*n* = 23), cystic fibrosis (*n* = 2), and advanced cardiac disease (*n* = 1). These patients could consequently not be included.

**Table 3:** Patient characteristics

Demographics	Responders (*n *= 150)	Non-responders (*n *= 150)
Male gender (*n*; %)	82 (54.7%)	76 (50.7%)
Age in years at operation (median with IQR)	2.7 (1.4–6.1)	2.7 (1.1–7.6)
Elapsed time since GP in years (median with IQR)	2.9 (1.4–4.7)	2.0 (0.8–5.0)
Patient deceased	0 (0.0%)	26 (17.3%)
Gastrostomy removed	14 (9.3%)	20 (13.3%)
Indication for GP		
Neurologic impaired development (n; %)	111 (74.0%)	106 (70.7%)
Cystic fibrosis	17 (11.3%)	12 (8.0%)
Cardiac disease	4 (2.7%)	6 (4.0%)
Gastrointestinal	4 (2.7%)	5 (3.3%)
Renal disease	5 (3.3%)	2 (1.3%)
Psychiatric/behavioral disease	3 (2.0%)	5 (3.3%)
Failure to thrive (undiagnosed)	4 (2.7%)	3 (2.0%)
Metabolic disorder	2 (1.3%)	3 (2.0%)
Dysmorphic facial features	0 (0.0%)	3 (2.0%)
Muscle disease	0 (0.0%)	2 (1.3%)
Lung disease	0 (0.0%)	2 (1.3%)
Esophagotracheal fistula due to foreign body	0 (0.0%)	1 (0.7%)

*GP* gastrostomy placement, *IQR* interquartile range

### Gastrostomy use and its complications

After GP, the gastrostomy was still in place in 87% of patients. Minor gastrostomy-related complications occurred in the majority of patients (90.7%), mainly consisting of hypergranulation (60.7%), infection of the gastrostomy site (48.7%), dislodgement of the catheter (43.3%), and obstruction of the catheter (23.3%). General satisfaction with the gastrostomy was graded as 8.2 (± 1.8) on a 10-point scale. After GP, some children did not tolerate feeding directly into the stomach and needed to be fed through a jejunal tube (*n* = 4; 3.4%).

### Health-related quality of life (HRQoL)

After GP, the mean total HRQoL score was 53.0 out of 100 (± 21.1). The mean psychosocial health summary score was 62.9 (± 34.0) and physical health summary score was 43.4 (± 17.2). Between the three subdomains of psychosocial health, the children scored best in the subdomain of emotional functioning (66.9 ± 17.5), followed by social functioning (64.9 ± 25.0), and school functioning (57.5 ± 24.7). These scores are all based on the observed data (*n* = 128).

Comparing our sample with the sample of Varni et al. [12] which we used as a reference standard (with a mean HRQoL score of 82.70 (SD 15.40)), we encountered a difference between the mean values of 29.7 points (95% CI 27.6–31.2).

**Fig. 2:**
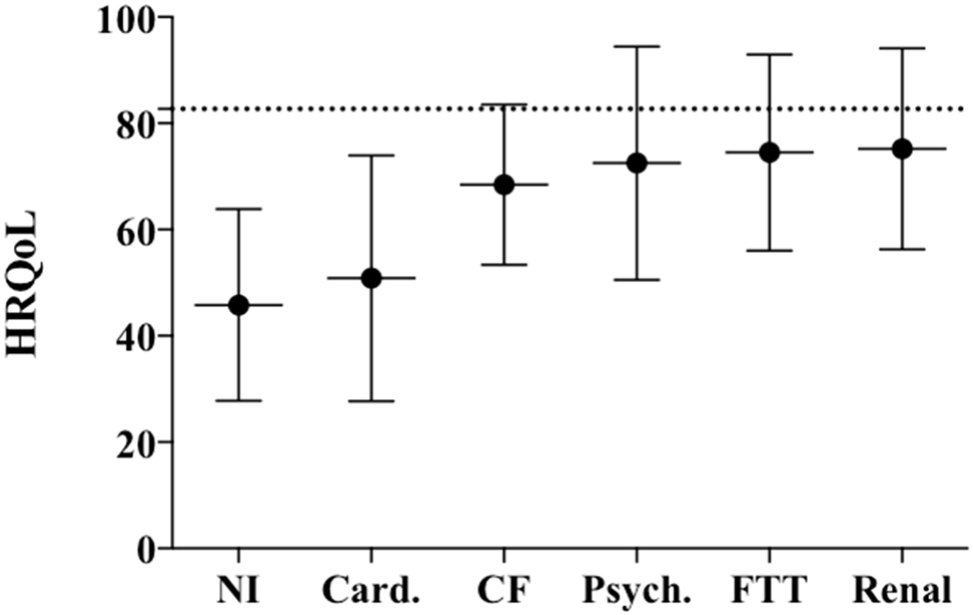
HRQoL stratified according to disease category. *HRQoL* health-related quality of life. Bars are depicted in means with standard deviations. The dotted line represents the mean HRQoL of a healthy child population, as measured by Varni et al. (82.70 ± 15.40). Neurologic impairment 45.8 (± 18.1); Cardiac disease 50.9 (± 23.1); Cystic fibrosis 68.5 (± 15.1); Behavioral disorder 72.5 (± 21.9); FTT Failure to thrive 74.5 (± 18.5); Renal disease 75.2 (± 18.9)

Figure 2 shows HRQoL after GP stratified according to our main categories of morbidity. Lowest HRQoL values were found in NI children (45.8 ± 18.1), followed by children with cardiac disease (50.9 ± 23.1), cystic fibrosis (68.5 ± 15.1), behavioral disorder (72.5 ± 21.9), failure to thrive (74.5 ± 18.5), and renal disease 75.2 (± 18.9).

### Predictors of HRQoL outcome after GP

All variables included in the multiple regression model of postoperative HRQoL and their coefficients are shown in Table 4. These results are based on the imputed data (*n* = 150).

**Table 4:** Results of the multiple linear regression analysis of health-related quality of life after gastrostomy placement (*n* = 150)

Predictors of health-related quality of life^a^	Mean difference (95% confidence interval)	Standardized beta coefficient	*p* value
Patient characteristics			
Age (years)	−1.2 (−2.3 to −0.2)	−0.23	0.03
Gender (female)	−5.5 (−16.3 to 5.3)	0.06	0.32
Follow-up time (years)	−0.2 (−3.8 to 3.5)	−0.16	0.93
Medical history			
Neurologic impairment	−21.4 (−32.6 to −10.3)	−0.58	< 0.001
Cardiac disease	−19.0 (−32.1 to −5.9)	−0.20	0.01
Previous gastrointestinal surgery	−15.2 (−28.2 to −1.7)	−0.12	0.30
Gastroesophageal reflux	−4.3 (−13.5 to 5.0)	−0.20	0.37
Acid exposure time (%) on 24-h pH monitoring	−0.4 (−1.5 to 0.7)	−0.08	0.48
Gastrostomy-related factors			
Jejunal feeding	−33.0 (−9.3 to −56.7)	−0.3	0.01
Reinterventions in operating theater	−16.2 (−33.2 to 0.8)	−0.2	0.06

^a^Negative predictors are correlated with lower outcome in health-related quality of life

Patients with NI had a significantly lower postoperative HRQoL compared to the rest of the patients with normal neurodevelopment, with a mean of 46.4 (± 18.2) in NI children compared to 71.4 (± 15.9) in NN children (confidence interval of the difference 23.8–26.5). Furthermore, NI had the highest predictive value of lower HRQoL outcome out of all variables included in the multivariate analysis, with an adjusted mean difference of 21.4 points (32.6–10.3) between NI and NN children. In subdomains of HRQoL, NI children scored particularly lower in the domain of physical health, with a mean physical health score of 27.5 (± 27.6) in NI children versus 71.3 (± 21.2) in NN children, with a mean difference of 43.8 points (41.8–45.9). In the psychosocial domain, NI children had a smaller disadvantage compared to NN children, with a mean psychosocial health score of 56.8 (± 17.2) in NI children compared to 70.6 (± 15.5) in NN children, with a mean difference of 13.7 points (12.4–15.1).

Children with cardiac disease had lower HRQoL after GP, with a mean difference of *−*19.0 points (−32.1 to −5.9) between affected and unaffected children. In addition, children with a history of gastrointestinal surgery (mean difference of −15.2 points, (−28.2 to −1.7)) and older patients at time of operation (−1.2 points per year; (−2.3 to −0.2)) had lower HRQoL.

The need for jejunal tube feeding through the gastrostomy site was also a predictor of lower HRQoL, with a mean difference of −33.0 between children with and children without the need for jejunal tube feeding (−9.3 to −56.7).

The other variables included in the analysis did not have a statistically significant predictive value on postoperative HRQoL.

## Discussion

This was the first study to investigate HRQoL in children with a gastrostomy using validated questionnaires. We found that after a mean follow-up time of 4.0 years (IQR 2.9–6.2), children with a gastrostomy had significantly lower HRQoL compared to the HRQoL of a large population of healthy normal children. Although it was suspected that HRQoL would be affected in this group of children, this had not previously been demonstrated.

After performing multiple regression analysis, we were able to evaluate parameters associated with lower HRQoL. NI was the main predictor of low HRQoL outcome. No data on health-related quality of life in specific comorbidities are available. However, our findings are in line with findings of another study by Varni et al. reporting on HRQoL assessment in 2500 children among 33 different disease categories. [12] They reported that NI children had the lowest HRQoL out of all disease categories, with a mean HRQoL of 66.85 (± 16.73) in their sample of 245 NI children compared to 82.70 (± 15.40) in their healthy sample. The mean HRQoL in our NI cohort was even lower than that of NI children in the population of Varni et al. This difference may be related to the severity of NI in our study population of children in need of a gastrostomy tube. However, because we do not possess the raw data of the Varni population, we cannot elaborate on this difference with any certainty.

The presence of cardiac disease was also a significant predictor of lower HRQoL after GP in our study, but to a lesser extent than NI. Other morbidity groups were not related to lower outcome in HRQoL in our study.

With respect to patient characteristics, we found that age at the time of operation was a predictor of lower HRQoL, indicating that older children undergoing GP are prone to have lower HRQoL in the long term. The cause of this relation is unclear. We found no significant differences in characteristics between children underneath 12 years of age and the adolescents (12–18 years) in our study group. A study performed by Mahant et al. found that children with progressive neurologic disorder had significantly lower HRQoL over time. [17] This may possibly explain the results in our study cohort. Another possible explanation is that the older a patient is at the time of GP, the more difficult it is to adjust to living with a gastrostomy. A prospective study following children with GP into adulthood is required to provide more certainty on this matter.

We investigated gastrostomy-related complications and their correlation with HRQoL. In a previous study, our research group found that a large number of children experience post-GP complications. While the severity of the complications is often minor, they are nevertheless often recurrent and sometimes require reintervention in the OR. [2] These complications may thus have a major impact on everyday life. The impact of these complications on HRQoL of children undergoing GP had never been investigated. In our study, GP-related complications requiring reintervention in the operating room were negatively correlated with HRQoL. Surprisingly, this association was not statistically significant (*p *= 0.06) indicating that GP-related complications may have only limited negative influence on HRQoL. Another possible explanation may lie in the long follow-up period of our study of 4.0 years (IQR 2.9–6.2). Since most complications occur in the first year after GP, the influence of these complications on HRQoL may diminish over time during follow-up.

A limitation of our study is that it is cross-sectional; it is therefore not possible to determine a causal relationship between GP and HRQoL. However, with multiple regression analysis we were able to identify parameters associated with lower or higher HRQoL. The study can be a good starting point for future prospective, longitudinal studies on the influence of GP on HRQoL.

Another limitation of this study is our response rate of 50%, which may have caused a bias in the selection of included patients. Furthermore, we encountered missing data in the 14.7% of the included children, mainly in the domain of psychosocial functioning and specifically the subdomain of ‘functioning at school/day care.’ Because of the severity of the underlying disease, caregivers were unable to answer these questions, even though the PedsQL^TM^ is validated for all different disease categories.

The current study provides insight into the characteristics of children with gastrostomy and the influences of patient-related characteristics and GP-related factors on their HRQoL. After GP, children have significantly lower HRQoL compared to a healthy pediatric population. Neurologic impairment, cardiac disease, a history of other gastrointestinal surgery, older age, and the need for jejunal feeding through the gastrostomy were predictive of even lower HRQoL. Data on HRQoL after GP in pediatric patients are important for treating physicians when children are referred for GP, especially in providing information to patients and their caregivers.
